# Sensory, textural, physico-chemical and enzymatic characterization of melted cheese with added potato and carrot peels

**DOI:** 10.3389/fnut.2023.1260076

**Published:** 2024-01-09

**Authors:** Ovidiu Tiţa, Maria Adelina Constantinescu, Mihaela Adriana Tiţa, Cristina Bătuşaru, Ion Mironescu

**Affiliations:** ^1^Department of Agricultural Sciences and Food Engineering, Lucian Blaga University of Sibiu, Sibiu, Romania; ^2^Academy of Land Forces “Nicolae Bălcescu” of Sibiu, Sibiu, Romania

**Keywords:** melted cheese, potato peel, carrot peel, sustainability, food waste

## Abstract

Food waste is one of the biggest societal problems in the globe due to its detrimental consequences on the environment. According to estimates from the Food and Agriculture Organization, this comes to about 1.3 billion tonnes per year. The current study aims to produce sustainable food products with high nutritional value by incorporating food waste. For the extraction of economically relevant products such as dietary fibers, biopolymers, natural antioxidants, and food additives, potato and carrot peel represent an inexpensive, valuable, and conveniently available resource. Cheese is a functional dairy product that people eat for its high nutritional content, which aids in the treatment of conditions including diabetes, obesity, hypertension, and digestive problems in addition to giving them energy. Thus a control sample and 10 samples of melted cheese with various amounts of potato and carrot peelings were prepared. To explore the nutritional value of potato and carrot peels in melted cheese, an analysis was conducted on the fluctuation of physicochemical (acidity, pH, dry matter, water activity, and salt content) and enzymatic (L-lactic acid, lactose, D-glucose, and D-galactose) parameters. Consumer acceptability of the products was assessed by textural and sensory analysis. During the whole storage period, the samples of melted cheese with potato and carrot peels recorded higher values than the control sample, the results obtained for them being better. Samples with added potato or carrot wastes were more stable over time, as compared to control samples.

## 1 Introduction

Due to its negative effects on the environment, food waste is a major social issue worldwide (1–4). The most important negative effects of food waste relate to the emission of 8–10% of greenhouse gases (5), rotting food produces methane (6) and in developed countries affects pricing policy (7). Across Europe, up to 50% of edible food is thrown away every year in households, supermarkets, restaurants, and throughout the food chain, and globally almost one billion people suffer from hunger and malnutrition (7). The European Union produces about 700 million tonnes of agricultural waste each year. Every year, roughly a third of the food produced worldwide is lost or wasted from farmers to consumers. The Food and Agriculture Organization (FAO) estimates that this amounts to around 1.3 billion tonnes annually (8). Vegetable waste is indiscriminately wasted without analyzing its biochemical composition and food value. A multitude of studies have shown that vegetable waste is rich in macro- and micro biomolecules. Due to their nutritional value, their use as ingredients in the development of new nutritional recipes is recommended (9). The recovery of vegetable food waste is an excellent opportunity for savings and sustainability. Industrial ecology has evolved significantly in recent years, with the primary goal being a “zero waste” society and economy where trash is repurposed as a raw material for new goods and applications (10).

Statistics from the FAO show that in 2016, more than 300 million tonnes of potatoes were produced annually (11). As a byproduct of the food processing industry, potato peel is a cheap, valuable, and easily accessible resource for the extraction of economically significant products, such as dietary fibers, biopolymers, natural antioxidants, and food additives (12, 13). Every year, between 70 and 140 thousand tons of peels are produced worldwide via industrial processing (14). Traditionally, potato peel waste has been utilized to make animal feed, fertilizer, or as a fuel for biogas, wasting minerals with anti-inflammatory, antioxidant, antibacterial, and apoptotic effects (14). It is determined that using potato peel trash is equivalent to turning iron into gold, which will benefit poor nations’ economies. Appropriate use of agricultural wastes must be made effective in maintaining high economic expansion and reducing poverty to strengthen the economies of developing nations (11). Creating by-products for the food industry out of plant waste can be a great way to increase economic growth and valorization.

Popular root vegetables like carrots have high levels of the antioxidants carotenoids, anthocyanidins, and saponins (9, 15, 16). By strengthening the immune system, these substances aid in the prevention of major diseases. Carrot peel is a significant by-product produced during the processing of carrot roots and is thought to be a large source of waste (an estimated 5.28 million tonnes annually), which raises the possibility of environmental damage (17). Potato peel is an excellent source of antioxidant compounds, mineral vitamins and phytochemicals (10, 18). Carrots are an excellent source of bioactive compounds, fiber and minerals (19). The capacity to decrease LDL oxidation, have anti-inflammatory characteristics, reduce oxidative stress, and enhance immunological function make carrot waste a valuable source of natural carotenoids that can be used to improve food items (20).

The finest way to consume cheese is fresh or matured. When necessary, dairy products including milk powder, whey, butter, and cream are added to such items at high temperatures with emulsifying salts to make a mixture known as melted cheese. Melted cheese can also be produced by pasteurizing curd, one or more types of cheese directly, or curd and another product (21–23). Cheese is a functional dairy product that is consumed for its high nutritional value, which helps prevent ailments including hypertension, diabetes, obesity, and digestive issues as well as providing energy (24–26).

The objective of the present study was to obtain sustainable food products with high nutritional value using potato and carrot waste. Due to the valuable nutritional properties that carrot and potato peels have, their valorization in food products is an excellent way to improve the final product and to transform food waste into a value added product. Thus a control sample and 10 samples of melted cheese with various amounts of potato and carrot peelings were prepared. The products were described in terms of their physicochemical and enzymatic properties as well as their consumer acceptability. The sample analysis period was just after obtaining and over 2 months.

## 2 Materials and methods

### 2.1 Drying kinetics of carrot and potato peels

Carrot peels were taken from a local company that deals with packaging and distribution of cleaned vegetables. The carrots were purchased from Top Fresh Handel B.V., country of origin the Netherlands. The potatoes were purchased from a local hypermarket, country of origin Romania, producer Agroprod Cartof SRL.

Drying of potato and carrot peels was done in an oven, manufacturer Electronic April at 50°C (9, 17). Every hour the moisture content was checked using a moisture analyzer, manufacturer AnD model ML-50 at 105°C. The drying process was set to reduce water content below 5%, according to the literature (9, 17). This implied drying carrot and potato peel during 16 and 24 h, respectively. Supplementary Figure 1 shows images of potato and carrot peels before and after drying. After drying, the samples were ground using a Heinner model HCG-150P coffee grinder for 15 s.

### 2.2 Manufacture of melted cheese

The melted cheese was processed under laboratory conditions according to a technological recipe developed by the research team using as components: cow’s curd (333 g), sheep’s curd (316 g), butter (148 g), skimmed milk powder (32.5 g), melting salt (30 g) and the addition of carrot and potato peel. The quantities mentioned were used to prepare one kilogram of melted cheese. The ingredients used were weighed, ground and mixed (except potato and carrot peel). The mixture was placed in a double boiler where it was melted at a temperature of 75°C. After melting took place homogenizing the mixture using a blender and then adding set proportions of carrot peel and potato peel. Depending on the amount of addition used, eleven samples of melted cheese were obtained as follows:

• CS: control sample (melted cheese without potato or carrot peel)
• MCC 0.5: melted cheese with carrot peel (0.5 g: 100 g)
• MCC 1: melted cheese with carrot peel (1 g: 100 g)
• MCC 1.5: melted cheese with carrot peel (1.5 g: 100 g)
• MCP 0.5: melted cheese with potato peel (0.5 g: 100 g)
• MCP 1: melted cheese with potato peel (1 g: 100 g)
• MCP 1.5: melted cheese with potato peel (1.5 g: 100 g)
• MCCP 0.5 0.5: melted cheese with carrot peel (0.5 g: 100 g) and potato peel (0.5 g: 100 g)
• MCCP 1 1: melted cheese with carrot peel (1 g: 100 g) and potato peel (1 g: 100 g)
• MCCP 1.5 1.5: melted cheese with carrot peel (1.5 g: 100 g) and potato peel (1.5 g: 100 g)
• MCCP 1.5 1: melted cheese with carrot peel (1.5 g: 100 g) and potato peel (1 g: 100 g)

**FIGURE 1:**
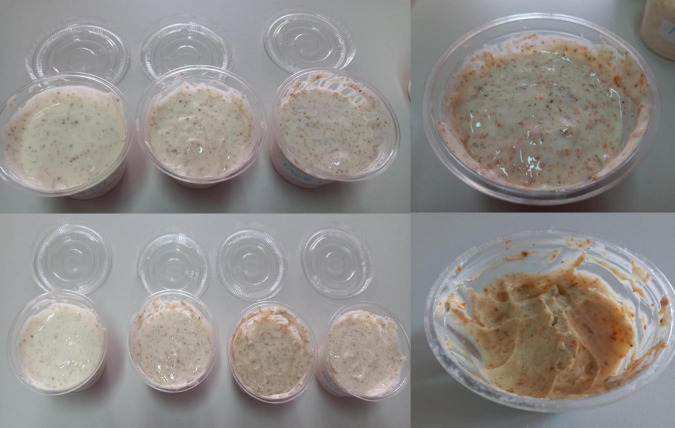
Melted cheese samples.

After the sensory study, making the MCCP 1.5 1 because the tasters gave the MCC 1.5 and MCP 1 samples the highest ratings. Images of the melted cheese products are displayed in Figure 1. The cheese products were packed in a plastic container with a lid and stored in the refrigerator at a temperature of 6°C.

### 2.3 Physicochemical determinations

#### 2.3.1 Acidity

A total of 10 grams of sample were homogenized with 5 ml distilled water and 1 ml phenolphthalein 2%. The homogeneous paste was titrated with 0.1N sodium hydroxide until a pink color appeared and it was maintained for 1 min. The acidity of the cheeses, expressed in Thörner degrees, is calculated with the formula (1) (27). Three repetitions were made for each sample.


(1) $$Acidity=\frac{V}{m}\cdot 100{[}^{{}^{\circ}}T]$$

Where:

V- volume of sodium hydroxide solution used for titration in cm^3^

m - the mass of the product taken as a working sample in grams.

#### 2.3.2 pH

The sample for analysis is obtained by mixing about 5 g of cheese with 5 ml of distilled water. The determination was performed using the Orion 2-Star pH meter, manufacturer Thermo Scientific by inserting the electrode into the sample to be analyzed and reading the pH value directly on the scale of the instrument (28). Three repetitions were made for each sample.

#### 2.3.3 Water activity

For the determination of water activity was used the LabMaster-aw, manufacturer Novasina AG at 25°C (29). Three repetitions were made for each sample.

#### 2.3.4 Dry matter content

Evaporation of water from the sample was carried out using the moisture analyzer, manufacturer AnD ML-50 at 150°C. Two grams of sample were placed on the moisture analyzer plate, and then water evaporation was started (30, 31). Three repetitions were made for each sample.

#### 2.3.5 Salt content

Over 5 grams of sample, 30 ml of warm distilled water (70–80°C) was added and transferred to a flask measured by 100 cm^3^. The contents of the flask were cooled and then brought to the mark with distilled water. After vigorous shaking there is a 15-min rest, followed by filtration. To 50 ml of filtrate add 1 ml of 1% potassium chromate, then titrate with 2.906% silver nitrate until copper-red staining is achieved. The sodium chloride content, expressed in grams of sodium chloride (NaCl) per 100 g of product, is calculated with the formula (2) (27). Three repetitions were made for each sample.


(2) $$\mathrm{Sodium}\,\mathrm{chloride}=\frac{V\cdot 100}{m\cdot V_{1}}\,\left[g/100\,g\,\mathrm{product}\right]$$

Where:

V- volume of silver nitrate solution used in the titration, in cm^3^

V_1_ - volume of product taken for analysis, in cm^3^

m - mass of product taken for analysis, in grams.

### 2.4 Enzymatic determinations

Enzyme kits, manufacturer R-Biopharm (Darmstadt, Germany), were used for enzyme assays. The Stat Fax 1904+ analyzer, manufacturer Awareness Technology, was used to measure the absorbance at 340 nm for cheese products (32). Three repetitions were made for each sample.

#### 2.4.1 L-lactic acid content

The procedure for sample preparation for analysis was carried out by the method R-Biopharm, (33) Cat. No. 10 139 084 035 and ISO 69B (1987) and ISO 9069 (1986) [(32); L-lactic acid].

#### 2.4.2 Lactose content

The procedure for sample preparation for analysis was carried out by the method R-Biopharm, (34) Cat. No. 10 176 303 035 and IDF 79B (1991) and ISO 5765-2 (1999) [(32); Lactose / D-Galactose].

#### 2.4.3 D-glucose content

The procedure for sample preparation for analysis was carried out by the method R-Biopharm, (35) Cat. No. 10 986 119 035 and IDF 79B (1991) and ISO 5765-1 (1999) [(32); Lactose / D-Glucose].

#### 2.4.4 D-galactose content

The procedure for sample preparation for analysis was carried out by the method R-Biopharm Cat. No. 10 176 303 035 and IDF 79B (1991) and ISO 5765-2 (1999) [(32); Lactose / D-Galactose].

### 2.5 Textural analysis

The TA.XT*puls*C texture analyzer from Stable Micro Systems was used to examine the texture of samples of melted cheese at 25°C. The analyzer is equipped with specialized software for texture analysis. A probe in the form of a disc was used to extrude the product up and around the edge of the disc. One repetition was made for each sample. Following the texture analyses, two textural characteristics are measured: firmness and consistency (36).

### 2.6 Sensory test

A group of seven non-trained panel tasters who frequently eat melted cheese performed sensory analysis on samples of melted cheese. The tasting was carried out on the first day, 15th day, 30th day, 45th day and 60th day of storage of the melted cheese products. The processing of the obtained results was performed using a non-numerical method based on several multi-person approval criteria described by Fadhil et al. in 2017 and 2020 (36–38).

The characteristics of the melted cheese products that were followed in the sensory analysis were consistency, viscosity, color, taste and smell. Table 1 represents the rating scale used in the collection of tasters’ opinions. Table 2 represents the level of importance of the criteria based on the scale.

**TABLE 1:** Linguistic assessment scale (37, 38, 60).

Scale	Description	Abbreviation
1	Like very much	LV
2	Like moderately	LM
3	Like slightly	LS
4	Neither like nor dislike	NT
5	Dislike slightly	DS
6	Dislike moderately	DM
7	Dislike very much	DV

**TABLE 2:** Criteria importance level (37, 38, 60).

Scale	Description	Abbreviation
1	Very high	LV
2	High	LM
3	Neither like nor dislike	NT
4	Low	DM
5	Very low	DV

The evaluation scale and importance level of the criteria were defined, and a matrix of evaluation criteria was created based on the opinions of the assessors and the chosen alternatives. The denial importance degree of the criteria was calculated using Formula (3).


(3) $$N\,e\,g\,\left(W_{k}\right)=\left(W_{q-k+1}\right)$$

where:

Neg (Wk) = negation of criteria k;

k = index;

q = scale amount.

For the approval process based on criteria, Formula (4) was used.


(4) $$V_{i\,j}=\min\left[N\,e\,g\,\left(W_{a\,k}\right)\vee V_{i\,j}\,\left(a_{k}\right)\right]$$

where:

V_*ij*_ = alternative i by person j;

V_*ij*_ (ak) = alternative i by person j on criteria k;

k = 1, 2, …, m.

Formula (5) was used to determine the value weights.


(5) $$Q_{k}=I\,n\,t\,\left[1+\left(k\cdot \frac{q-1}{r}\right)\right]$$

where:

Q_*k*_ = score k;

Int = integer;

r = number of assessors.

Formula (6) was used to determine the tasting process of the tasters.


(6) $$V_{i}=f\,\left(V_{i}\right)\,\max\left[Q_{i}\,\wedge \,b_{j}\right]$$

where:

V_*i*_ = total score for alternative i;

Q_*i*_ = score j;

j = 1, 2, …, m;

b_*j*_ = order from the biggest alternative score i from alternative score j (37–39).

### 2.7 Statistical significance of results

The results obtained from acidity, pH, water activity, dry matter, salt content and enzymatic analysis were statistically processed using Minitab software version 14. Analysis of variance (ANOVA) and Duncan’s multiple range test, applied at the 5% level of significance (*p* < 0.05), were used to compare the mean values and assess the results. Every measurement was done three times.

## 3 Results and discussion

### 3.1 Drying kinetics of carrot and potato peels

**FIGURE 2:**
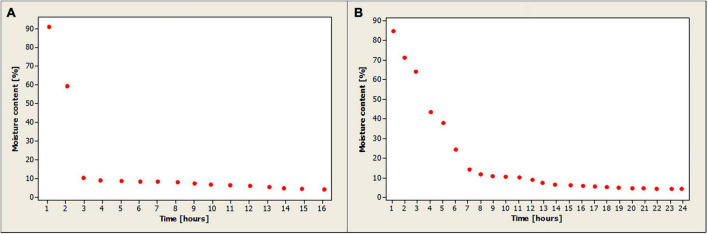
Evolution of moisture content in carrot peel **(A)** and potato peel **(B)**.

Carrot peel moisture content evolution is depicted in Figure 2A. The samples were dried at 50°C for 16 h. The moisture content of the fresh peel is 91%, also confirmed by Nguyen and Scarlett (40). In the first 2 h of drying, more than 83% of the water evaporates, with the moisture content reaching 10.3%. One can relate this intense evaporation to the intense action of hot air on the peel components, the main component being the plant cell wall, which serves as a mechanical support element and external barrier. The structure of plant tissues can greatly influence water removal as it affects the rate of mass transfer during drying (41). After the first 2 h of drying, the evaporation process has stabilized and this can be attributed to the peridermis (42) which limits water loss and forms a barrier for the absorption of chemicals (43). The final moisture content is 4.2%, which is in agreement with studies by Nguyen and Le (17) who report a final content of 6.86 ± 0.10 a g water/100 g dried carrot sample and by Hashmi et al. (9) who report a final content of 4.2%.

The progression of the moisture content in potato peels is depicted in Figure 2B. The samples were dried for 24 h at 50°C. Compared to the drying process of carrot peels, in this case, the evaporation of water is much more stable. In the first 2 h of drying about 25% of the water is lost, this can be influenced by the chemical composition of the leaves that protect and cover the tubers (44). Conventional drying is a time-consuming, energy-intensive process that, coupled with high temperatures, can lead to undesirable changes such as nutrient degradation or poor rehydration capacity (45). To ensure as little loss of nutrient compounds as possible, it was decided that drying should also be carried out at 50 degrees, a fact confirmed by Soltan et al. (46). The final moisture content is 4.2%, which is in agreement with the studies carried out by Pérez-Chabela et al. (47) who obtained a moisture content of potato peel flour of 4.95 ± 0.17% and by Hashmi et al. who obtained a moisture content of 4.95% ± 0.17% (9).

### 3.2 Physicochemical determinations

#### 3.2.1 Acidity

Acidity changes in melted cheese products during 8 weeks of storage at 4°C are shown in Supplementary Table 3. During the 60 days of storage, it can be observed that the acidity of all samples increases at the same time as the pH decreases. Since the supplemented samples are more acidic than the control sample, the effect of adding carrot and potato peel is seen. Because there are no phenolic antimicrobial compounds in processed cheese containing carrot and potato peel their acidity has increased to a lesser extent than in plain cheese (15).

The increasing trend of the acidity of the samples is directly proportional to the amount of addition. Cheese products with added potatoes show a similar increase as samples with added carrots. The acidity of the samples with mixed addition shows similar values to those of the samples with a single addition. A significant difference is found in sample MCC 1 1 which differs greatly from the results of the other samples during the whole storage period. This may be due to acids in the peels of catnips and carrots (21), but also to microbial growth (25). During the first 15 days of storage, the increase in acidity is relatively slow compared to the rest of the period where the increase is higher. Bandyopadhyay et al. (48) confirm the increase in acidity of carrot paste samples over 20 days of storage. The acidity of the pizza cheese decreases with the increase in the content of soybean oil and the decrease in the content of carrot extract (25).

#### 3.2.2 pH

pH changes in melted cheese products during 8 weeks of storage at 4 °C are shown in Supplementary Table 3. During the 60 days of storage, for all samples, the pH decreases while acidity increases. The gradual decrease in pH in cheese is due to the production of lactic acid by lactic acid bacteria (15). The effect of carrot and potato addition is present, with the added samples having a lower pH compared to the control sample over the entire 60-day period. Acids present in potato and carrot peel influence the pH and acidity of samples (21).

The amount of addition in the sample influences the pH, with 0.5 g samples having a higher pH compared to 1.5 g samples. The mixed-added samples record values close to those of the single-added samples. During the first 15 days, there is a slow decrease and after this day the decrease accelerates. Bandyopadhyay et al. (48) confirm the decrease in pH of carrot paste samples over 20 days of storage. Sharifi et al. (15) confirmed a pH decrease and acidity increase for yogurt samples with carrot extract for 28 days. In acid-fermented products the amount of acid increases during the storage period, which is called excessive acidification or subsequent acidification, which is caused by the enzyme β-galactosidase which remains at 0–5°C.

#### 3.2.3 Water activity

Water activity in melted cheese products during 8 weeks of storage at 4 °C is shown in Supplementary Table 3. During the 60 days of storage, the water activity of the control sample increases, while that of the added samples decreases. The evolution over time is significantly similar, being more stable in the case of shell samples. The amount of added peel influences water activity, which decreases as the amount of added peel increases. It is well known that foods with high water activity (aw > 0.6) and high moisture content are very susceptible to microbial spoilage and contamination (49).

The results obtained are confirmed by Emam (50) showing that the level of added starch resulted in a significant impairment of water activity in cheese with 2.5% potato starch obtaining the lowest water activity of all samples. López-Córdoba (49) and Szafrańska et al. (51) confirm that the water activity does not have a significant evolution during the storage period, the addition not strongly influencing the results obtained.

#### 3.2.4 Dry matter content

Dry matter content in melted cheese products during 8 weeks of storage at 4 °C is shown in Supplementary Table 3. During storage dry matter growth occurs for all samples. The results obtained are significantly similar in the first 45 days, the evolution being stronger after this day. Carrot and potato peels influence the dry matter of the samples, with the results obtained on the first day of analysis being higher compared to the control sample. However, stability of dry matter growth is observed for samples with addition. In the study by Kamel et al. (19) the moisture content of soft cheese products with carrots increases during the storage period. The sample with the highest carrot content recorded the highest moisture values throughout the storage period. Adding soybean oil to pizza cheese decreases the moisture content during storage (25).

#### 3.2.5 Salt content

The salt content in melted cheese products during 8 weeks of storage at 4°C is shown in Supplementary Table 3. During the 60 days of storage, the salt content decreases in all samples analyzed. The results obtained are significantly similar for all cheese products, with a stable decrease process. As with the other analyses, the effect of carrot and potato peels is present, as these samples have a low salt content compared to the control sample. As can be seen, the salt content of carrots and potato peels does not influence the total content. The carrot peel samples have a higher salt content compared to the potato peel samples. The samples with mixed addition have significantly similar values to the samples with a single addition. The MCCP 1 1 sample has the lowest salt content, the result being significantly different from the other samples.

Kamel et al. (19) show that in the case of the samples of soft cheese with carrots, the salt content increases until the 14th day of storage, and then starts to decrease until the 21st day of storage. Also, the salt content is influenced by the way the cheese is stored. For the carrot cheese product analyzed immediately after production, the salt content is lower compared to the refrigerated sample (52).

### 3.3 Enzymatic determinations

#### 3.3.1 L-lactic acid content

L-lactic acid content in melted cheese products during 8 weeks of storage at 4°C is shown in Supplementary Table 4. During storage, the L-lactic acid content increases in all samples. The evolution in the case of the control sample is less stable than in the case of the added samples. The results obtained for carrot and potato peel samples are significantly similar throughout the storage period. The influence of addition is also visible in this analysis because the potato peels contain lactic acid (53, 54). The increased lactic acid content is also confirmed by acidity determination. On the first day of deposition, the control sample had the lowest L-lactic acid content, reaching the highest content on the 60th day. The MCCP 1 1 sample has the highest values compared to the samples with the addition.

#### 3.3.2 Lactose content

Lactose content in melted cheese products during 8 weeks of storage at 4°C is shown in Supplementary Table 4. The decrease in lactose content during the 60 days of storage is certified by the increase in L-lactic acid content. All samples record the highest lactose content on the first day of storage. The results are significantly similar for all samples, but it should be noted that the decrease is much more stable in the case of the added samples. Because potato and carrot peelings contain L-lactic acid (53, 54), the lactose content of cheese with peelings is lower than that of the control sample. If in the case of the samples with reduced addition of peel, the higher lactose content is recorded, we can observe that in the case of the MCCP 1 1 sample, the lowest content was obtained.

#### 3.3.3 D-glucose content

D-glucose content in melted cheese products during 8 weeks of storage at 4°C is shown in Supplementary Table 4. During the storage period, the D-glucose content of cheese products decreases, which is in line with the lactose content. The variation is significantly similar for all samples, the decrease being stable. The effect of carrot and potato peels is confirmed in this case as well, with the added samples recording higher values compared to the control sample. This is certified by the glucose content of the potato peel (12, 55). With the increasing amount of peels in the samples, the D-glucose content increases. The control sample has the lowest content. As can be seen, the D-glucose content of the samples is much lower compared to D-galactose which is found in a more significant proportion.

#### 3.3.4 D-galactose content

D-galactose content in melted cheese products during 8 weeks of storage at 4°C is shown in Supplementary Table 4. During the 60 days of storage, the D-galactose content of all samples decreases. The results obtained are significantly similar, their evolution being stable. The results obtained correlate with those from previous analyses, lactose, D-glucose and L-lactic acid content. The influence of carrot and potato peels is also visible in this case, the samples with the addition having a higher content compared to the control sample. Although potato peels do not have a significant content of D-galactose (56, 57), the results obtained are probably influenced by the content of melted cheese. The control sample has the lowest content, and the samples with the highest amount of peels have the highest.

### 3.4 Textural analysis

**FIGURE 3:**
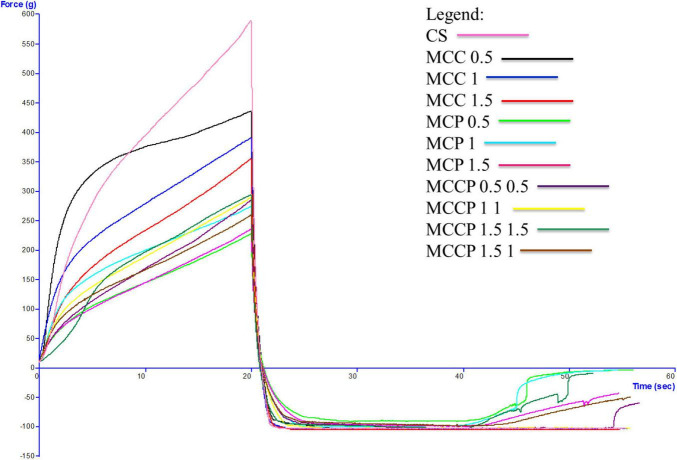
Evaluation of the textural characteristics of melted cheese samples on the first day of storage.

**FIGURE 4:**
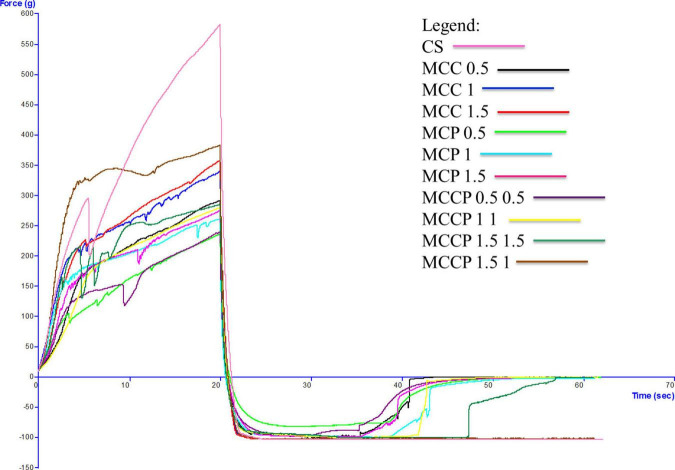
Evaluation of the textural characteristics of melted cheese samples on the 15th day of storage.

**FIGURE 5:**
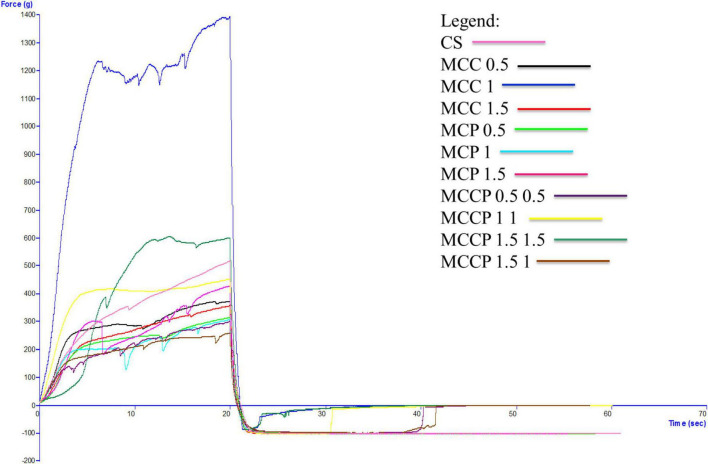
Evaluation of the textural characteristics of melted cheese samples on the 30th day of storage.

**FIGURE 6:**
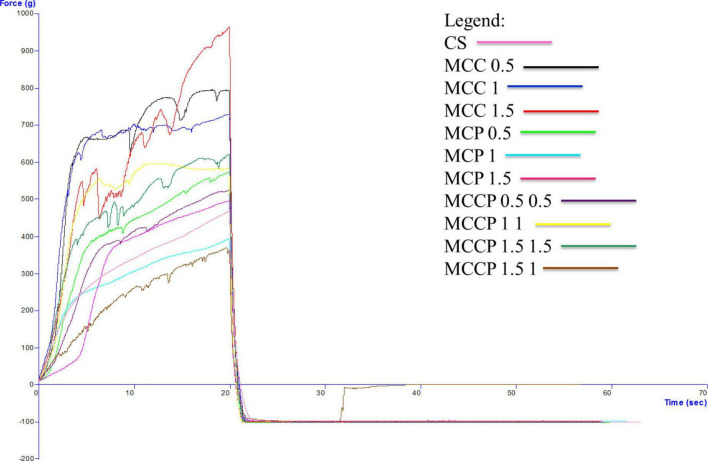
Evaluation of the textural characteristics of melted cheese samples on the 45th day of storage.

**FIGURE 7:**
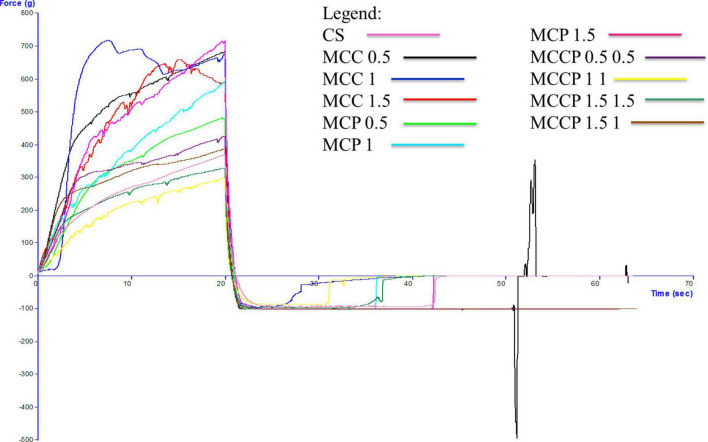
Evaluation of the textural characteristics of melted cheese samples on the 60th day of storage.

The evolution of the textural characteristics of the melted cheese in all days of storage is shown in Figure 3 (first day), Figure 4 (day 15), Figure 5 (day 30), Figure 6 (day 45), and Figure 7 (day 60). In the first 15 days of storage, firmness decreases in most samples, except for MCC 1.5, MCP 0.5, MCP 1.5, and MCCP 1.5 1, where the results are significantly similar. The decrease in this textural parameter can be attributed to an increase in moisture as well as a decrease in the protein content of the cheese (21). The various qualities of the additives used, which are reflected in the biochemical processes taking place during ripening and chilled storage, might be attributed to variations in the texture of the finished products. The highest firmness after ripening and refrigerated storage was observed for cheeses obtained with the addition of potato peel, while the lowest values of this parameter were recorded for cheeses with the addition of carrots (52). Between day 15 and the last day of storage, there is a strong evolution of firmness with increasing values recorded for most samples.

The influence of carrot and potato peel addition on firmness is present as these samples record low values compared to the control sample. The amount of carrot peel is inversely proportional to the firmness value. Firmness during storage may be related to the destruction of the microstructure of cheese products containing carrot peel, due to the enzymatic activity of carrot peel (58). In the study conducted by Mohammadi et al. (58), in the textural analysis of the whey-less cheese product with carrot powder, the hardness decreased during the 6 weeks. The control sample had the highest hardness values and the samples with carrot powder had lower values, being influenced by the amount of powder (58). In the textural analysis of pizza cheese with carrot extract (25), the textural desirability increased by decreasing the carrot extract content and increasing the soybean oil content (25). In the study conducted by Gustaw et al in 2020, it was demonstrated that the texture of carrot cheese is influenced by the storage method. The cheese product analyzed immediately after production had a higher hardness compared to the one analyzed after storage at refrigeration temperature (52).

The firmness of samples of cheese with potato peel is not significantly influenced by this addition, but its evolution over time is fairly stable. Up to day 45 of storage, the firmness value is lower compared to the control sample. The firmness of the cheese appeared to be enhanced and the curd network was strengthened by the interaction of phenolic chemicals with casein (58). This is also confirmed by Rafiq and Ghosh (21) who observed that an increase in potato incorporation tended to decrease hardness. In the study conducted by Spelbrink et al. in 2015 (59), the control sample had a much higher hardness compared to the cheese and potato products. The hardness of the potato samples decreased with the amount of addition (21). The results obtained for consistency are correlated with those obtained for firmness, with a similar trend of decreasing until day 15 and then increasing.

### 3.5 Sensory analysis

#### 3.5.1 Determining alternatives

In the initial stage, Formula (3) was used to calculate the negation of the importance level of the criteria, and each index k was used to derive the negation of the value of the criteria weight. Supplementary Table 1 displays the findings of the calculation of the criteria’s negation. Supplementary Table 2 displays the tasters’ opinions as determined by the distribution of the questionnaire.

#### 3.5.2 Determining the criteria

The findings for each alternative were as follows because, in the tester’s judgment, Formula (4) was used to calculate the acceptance requirements for each alternative. Table 3 contains the criteria for each alternative according to the tasters’ opinions.

**TABLE 3:** The criteria for each alternative according to the tasters’ opinion.

Period	Alternative	V_1_	V_2_	V_3_	V_4_	V_5_	V_6_	V_7_
Day 1	1	LM	LM	LM	LM	LM	LM	LM
	2	LM	LM	LM	LM	LM	LM	LM
	3	LM	LM	LM	LM	LM	LM	LM
	4	LV	LV	LV	LV	LV	LV	LV
	5	LM	LM	LM	LM	LM	LM	LM
	6	LV	LV	LV	LV	LV	LV	LM
	7	LM	LM	LM	LM	LM	LM	LM
	8	LM	LM	LM	LM	LM	LM	LM
	9	LM	LM	LM	LM	LM	LM	LV
	10	LM	LM	LM	LM	LM	LM	LM
Day 15	1	LS	LS	LS	LS	LS	LS	LS
	2	LM	LS	LM	LM	LM	LM	LM
	3	LS	LM	LS	LM	LM	LS	LM
	4	LV	LM	LM	LM	LM	LM	LM
	5	LS	LM	LM	LM	LM	LM	LM
	6	LV	LM	LM	LM	LV	LM	LM
	7	LS	LM	LM	LM	LS	LM	LM
	8	LS	LM	LM	LM	LM	LS	LM
	9	LM	LM	LM	LS	LS	LM	LM
	10	LS	LM	LM	LM	LS	LM	LM
Day 30	1	LS	LS	LS	LS	NT	LS	LS
	2	LS	LS	LS	LS	LS	LS	LS
	3	LS	LM	LS	LS	LS	LS	LM
	4	LM	LM	LM	LM	LM	LM	LM
	5	NT	LS	LS	LS	LS	LS	LS
	6	LM	LM	LM	LM	LM	LM	LM
	7	LS	LS	LS	LS	LS	LS	LS
	8	LS	LS	LM	LM	LS	LS	LS
	9	LS	LS	LS	LS	LS	LS	LM
	10	LS	LS	LM	LS	LS	LS	LM
Day 45	1	NT	NT	NT	NT	NT	LS	LS
	2	LS	LS	LS	LS	LS	LS	LS
	3	LS	LM	LS	LS	LS	LS	LS
	4	LM	LM	LM	LM	LM	LM	LM
	5	NT	LS	LS	LS	LS	LS	LS
	6	LM	LM	LM	LM	LM	LM	LM
	7	LS	LS	LS	LS	LS	LS	LS
	8	LS	LS	LS	LS	LS	LS	LS
	9	LS	LS	LS	NT	LS	LS	LS
	10	NT	LS	LS	LS	LS	LS	LS
Day 60	1	DS	NT	DS	DS	DS	NT	NT
	2	NT	NT	NT	NT	NT	LS	LS
	3	NT	LS	LS	NT	NT	NT	LS
	4	LM	LS	LS	LS	LS	LS	LS
	5	NT	NT	NT	NT	NT	LS	NT
	6	LM	LS	LS	LS	LS	LS	LS
	7	NT	NT	LS	NT	NT	LS	LS
	8	NT	NT	LS	NT	LS	LS	NT
	9	LS	LS	LS	NT	LS	NT	LS
	10	NT	LS	LS	NT	NT	NT	LS

#### 3.5.3 Determining the tasters’ approval process

Before calculating a taster’s aggregation process, Formula (5) was used to determine the value weights. The value weights for Q1, Q2, Q3, Q4, Q5, Q6, and Q7 are DM, DS, NT, NT, LS, LM, and LV, respectively. To determine the tasters’ aggregation process, Formula (6) was used. Table 4 contains the results of the determination of the tasters’ agreement process.

**TABLE 4:** Determining the tasters’ approval process.

Period	A1	A2	A3	A4	A5	A6	A7	A8	A9	A10
Day 1	LM	LM	LM	LV	LM	LM	LM	LM	LM	LM
Day 15	LS	LM	LS	LM	LM	LM	LS	LS	LS	LS
Day 30	LS	LS	LS	LM	LS	LM	LS	LS	LS	LS
Day 45	NT	LS	LS	LM	LS	LM	LS	LS	LS	LS
Day 60	NT	NT	NT	LS	NT	LS	NT	NT	LS	NT

On the first day of tasting, the best results were obtained by sample MCC 1.5, which was rated LV (Like very much) and sample MCP 1 which was rated LM (Like moderately). On the 15th day, the two samples also obtained the highest results, both achieving the LM (Like moderately) rating. On the 30th and 45th days, MCC 1.5 and MCP 1 were the only samples that were rated LM (Like moderately), the other samples were rated lower. On the last day of storage MCC 1.5, MCP 1, and MCCP 1 1 were rated LS (Like slightly) and the other samples were rated NT (Neither like nor dislike).

In the sensory analysis of a dairy dessert (rasgulla) with carrots conducted by Bandyopadhyay et al. in 2007, it was shown that the highest ratings were given on the first day of tasting (analysis period: 20 days), and the samples with the highest addition of carrots were the most appreciated (48). Motevalizadeh et al. (25) sensory-analyzed pizza cheese with soybean oil and carrot extract. The highest scores were obtained for the cheese product with a medium addition of carrot extract and a low addition of soybean oil (25). In another sensory research from 2023 of the probiotic soft cheese supplemented with carrot powder, it was concluded that the control sample obtained the highest qualifications, and the cheese products with the addition of carrot powder obtained lower results being influenced by the addition of the powder (19). In 2017 Rafiq and Ghosh conducted a study on processed cheese with added potato. Following sensory analysis, it was concluded that the samples with 20 and 30% addition performed better than the sample with 40% potato addition (21). In the study conducted by Spelbrink et al. in 2015, sensory analysis of cheese products was influenced by the addition of potato patatin. Samples with medium addition outperformed samples with high potato addition. Also, the results obtained after a storage period of 6 weeks were superior to those obtained after 13 weeks of storage (59).

## 4 Conclusion

The current study aimed to achieve sustainable food products with high nutritional value by incorporating ingredients obtained from food waste. To achieve the proposed objective, different samples of melted cheese with added potato peel and carrot peel were made. The physicochemical parameters of melted cheese were influenced by carrot and potato peel softening by increasing acidity, water and dry matter activity and by decreasing pH and salt content. The effect of the addition was present again by increasing the content of L-lactic acid and decreasing the content of glucose, lactose and galactose. The influence of the added peels in the melted cheese was analyzed also during storage, and the results obtained showed that the evolution of the physicochemical and enzymatic parameters in these samples was stable compared to the control sample. The textural and sensory properties of melted cheese were positively influenced by the two types of peel, based on the results obtained for these products. Consumer acceptability is very important to conclude whether a food product has the potential to be marketed. The results obtained showed an increased stability of the cheese samples with added cheese, which certifies the influence of carrot and potato peel.

The use of fruit and vegetable waste can be an important alternative in the food industry, leading to products with high nutritional value and reducing the large amount of waste affecting the environment. The products obtained in this study can be a suitable alternative for processors, contributing to the valorization of potato and carrot residues and the development of the economy especially for developing countries. However, the present study is a first step in the analysis of these types of products, which is why it is recommended to deepen the influence of the addition of carrot and potato peel on other parameters in melted cheese. The use of another type of food waste (e.g., tomato peel, onion peel), as well as another dairy product (e.g., yogurt, ripened cheese), can also be considered.

## Data availability statement

The original contributions presented in this study are included in this article/Supplementary material, further inquiries can be directed to the corresponding authors.

## Author contributions

OT: Conceptualization, Funding acquisition, Project administration, Supervision, Validation, Writing—review and editing. MC: Conceptualization, Data curation, Investigation, Software, Visualization, Writing—original draft. MT: Conceptualization, Methodology, Resources, Supervision, Validation, Writing—review and editing. CB: Data curation, Methodology, Visualization, Writing—original draft. IM: Data curation, Formal analysis, Software, Writing—review and editing.
