# Supplementary material for: Sensory, textural, physico-chemical and enzymatic characterization of melted cheese with added potato and carrot peels
Source: Front Nutr. 2024 Jan 9;10:1260076. doi: 10.3389/fnut.2023.1260076 (PMC10807040; doi:10.3389/fnut.2023.1260076)
Supplement: Supplementary file 1 [file Data_Sheet_1.docx]

Supplementary Material

Sensory, Textural, Physico-Chemical and Enzymatic Characterization of Melted Cheese with Added Potato and Carrot Peels

Ovidiu Tița*, Maria Adelina Constantinescu*, Mihaela Adriana Tița, Cristina Bătușaru, Ion Mironescu

*** Correspondence:** Ovidiu Tița: [ovidiu.tita@ulbsibiu.ro](mailto:ovidiu.tita@ulbsibiu.ro)

Maria Adelina Constantinescu: adelina.constantinescu@ulbsibiu.ro

# Supplementary Tables

**Supplementary Table 1.** Negation of criteria importance level (R Fadhil et al. 2017) (Rahmat Fadhil, Agustina, and Hayati 2020) (M. A. Tița et al. 2022)

| **Criteria Importance Level** | **The Negation of Criteria Importance Level** |
| --- | --- |
| Criteria 1 = Very high | Criteria 1 = Very low |
| Criteria 2 = High | Criteria 2 = Low |
| Criteria 3 = Neither like nor dislike | Criteria 3 = Neither like nor dislike |
| Criteria 4 = Low | Criteria 4 = High |
| Criteria 5 = Very low | Criteria 5 = Very high |

| **Person**  **Supplementary Table 2.** Criteria of assessment by each person of all alternatives | **Alternative** | **Criteria** | | | | | | | | | | | | | | | | | | | | | | | | |
| --- | --- | --- | --- | --- | --- | --- | --- | --- | --- | --- | --- | --- | --- | --- | --- | --- | --- | --- | --- | --- | --- | --- | --- | --- | --- | --- |
|  |  | **Consistency** | | | | | **Viscosity** | | | | | **Color** | | | | | **Taste** | | | | | **Smell** | | | | |
|  |  | Day 1 | Day 15 | Day 30 | Day 45 | Day 60 | Day 1 | Day 15 | Day 30 | Day 45 | Day 60 | Day 1 | Day 15 | Day 30 | Day 45 | Day 60 | Day 1 | Day 15 | Day 30 | Day 45 | Day 60 | Day 1 | Day 15 | Day 30 | Day 45 | Day 60 |
| P1 | A1 | LM | LS | LS | NT | DS | LM | LM | LS | LS | NT | LM | LS | LS | NT | NT | LM | LS | LS | NT | NT | LM | LS | LS | NT | DS |
|  | A2 | LM | LM | LS | LS | LS | LM | LM | LM | LS | LS | LM | LM | LS | LS | NT | LM | LM | LS | LS | LS | LM | LM | LM | LS | LS |
|  | A3 | LM | LM | LM | LS | LS | LM | LM | LM | LM | LS | LM | LS | LS | LS | NT | LV | LM | LM | LS | LS | LV | LM | LM | LS | LS |
|  | A4 | LV | LV | LM | LM | LM | LV | LV | LV | LM | LM | LV | LV | LV | LM | LM | LV | LV | LM | LM | LM | LV | LV | LM | LM | LM |
|  | A5 | LM | LS | LS | LS | LS | LM | LM | LM | LM | LS | LM | LS | NT | NT | NT | LM | LM | LS | LS | LS | LM | LM | LS | LS | LS |
|  | A6 | LV | LV | LM | LM | LM | LV | LV | LV | LM | LM | LV | LV | LV | LM | LM | LV | LV | LM | LM | LS | LV | LM | LM | LM | LM |
|  | A7 | LM | LM | LM | LM | LS | LM | LS | LS | LS | NT | LM | LM | LS | LS | LS | LM | LM | LM | LS | LS | LM | LM | LS | LS | LS |
|  | A8 | LM | LM | LS | LS | LS | LM | LM | LM | LS | LS | LM | LS | LS | LS | NT | LM | LM | LS | LS | NT | LM | LM | LS | LS | NT |
|  | A9 | LM | LM | LM | LM | LS | LM | LM | LM | LM | LS | LM | LM | LS | LS | LS | LM | LM | LS | LS | LS | LM | LS | LS | NT | NT |
|  | A10 | LM | LM | LS | LS | NT | LM | LM | LS | LS | LS | LM | LS | LS | NT | NT | LM | LM | LM | LS | LS | LM | LM | LS | LS | LS |
| P2 | A1 | LM | LM | LM | LS | LS | LM | LS | LS | NT | NT | LM | LM | LS | NT | NT | LM | LS | LS | NT | NT | LM | LS | NT | NT | DS |
|  | A2 | LM | LM | LS | LS | NT | LM | LM | LS | LS | LS | LM | LS | LS | LS | LS | LM | LM | LS | LS | NT | LM | LM | LS | LS | NT |
|  | A3 | LV | LM | LM | LM | LS | LM | LM | LM | LM | LS | LM | LM | LM | LM | LS | LV | LM | LM | LS | LS | LV | LM | LS | LS | LS |
|  | A4 | LV | LV | LM | LM | LM | LV | LV | LM | LM | LM | LV | LM | LM | LM | LS | LV | LV | LM | LM | LM | LV | LV | LM | LM | LM |
|  | A5 | LM | LM | LM | LS | LS | LM | LM | LS | LS | NT | LM | LM | LS | LS | LS | LM | LM | LS | LS | NT | LM | LS | LS | LS | NT |
|  | A6 | LV | LM | LM | LM | LS | LV | LV | LM | LM | LM | LV | LV | LM | LM | LS | LV | LM | LM | LM | LS | LV | LM | LM | LS | LS |
|  | A7 | LM | LM | LS | LS | NT | LM | LM | LS | LS | LS | LM | LM | LM | LS | LS | LM | LM | LM | LS | LS | LM | LM | LS | LS | LS |
|  | A8 | LM | LM | LS | LS | NT | LM | LM | LM | LS | LS | LM | LM | LM | LS | LS | LM | LM | LS | LS | NT | LM | LS | LS | LS | NT |
|  | A9 | LM | LM | LM | LS | LS | LM | LM | LM | LS | LS | LM | LM | LS | LS | LS | LM | LM | LM | LS | LS | LM | LM | LS | LS | LS |
|  | A10 | LM | LM | LM | LM | LS | LM | LM | LS | LS | LS | LV | LM | LM | LS | LS | LM | LM | LS | LS | LS | LM | LS | LS | NT | NT |
| P3 | A1 | LM | LM | LS | LS | NT | LM | LS | LS | NT | DS | LM | LM | LS | LS | NT | LM | LS | LS | NT | NT | LM | LS | NT | NT | DS |
|  | A2 | LM | LM | LS | LS | LS | LM | LM | LM | LS | LS | LM | LM | LS | LS | NT | LM | LM | LS | LS | LS | LM | LM | LS | LS | NT |
|  | A3 | LM | LM | LM | LS | LS | LM | LS | LS | LS | LS | LM | LM | LM | LS | LS | LV | LM | LM | LS | LS | LV | LM | LM | LM | LS |
|  | A4 | LV | LV | LM | LM | LM | LV | LM | LM | LM | LS | LV | LV | LM | LM | LM | LV | LV | LM | LM | LM | LV | LV | LM | LM | LM |
|  | A5 | LM | LM | LS | LS | LS | LM | LM | LS | LS | NT | LM | LM | LM | LS | NT | LM | LM | LM | LS | LS | LM | LM | LS | LS | LS |
|  | A6 | LV | LM | LM | LM | LS | LV | LV | LM | LM | LS | LV | LV | LM | LM | LM | LV | LM | LM | LM | LM | LV | LV | LM | LM | LM |
|  | A7 | LM | LM | LM | LS | LS | LM | LM | LM | LM | LS | LM | LM | LS | LS | LS | LM | LM | LS | LS | NT | LM | LM | LS | LS | NT |
|  | A8 | LM | LM | LM | LS | LS | LM | LM | LM | LS | LS | LM | LM | LM | LM | LS | LM | LM | LS | LS | LS | LM | LM | LM | LS | LS |
|  | A9 | LM | LM | LM | LS | LS | LM | LM | LM | LS | LS | LM | LM | LS | LS | LS | LM | LS | LS | LS | LS | LM | LS | LS | NT | NT |
|  | A10 | LM | LM | LM | LM | LS | LM | LM | LM | LS | LS | LV | LM | LM | LS | LS | LM | LM | LS | LS | LS | LM | LM | LM | LS | LS |
| P4 | A1 | LM | LS | LS | NT | DS | LM | LM | LS | LS | NT | LM | LS | LS | LS | NT | LM | LM | LS | LS | LS | LM | LM | LM | LS | LS |
|  | A2 | LM | LM | LS | LS | NT | LM | LM | LS | LS | NT | LM | LM | LS | LS | LS | LM | LM | LM | LS | LS | LM | LM | LM | LS | LS |
|  | A3 | LM | LM | LM | LS | LS | LM | LM | LS | LS | LS | LM | LM | LS | LS | NT | LM | LM | LS | LS | LS | LM | LM | LS | LS | LS |
|  | A4 | LV | LV | LM | LM | LM | LV | LM | LM | LM | LS | LV | LV | LM | LM | LS | LV | LV | LM | LM | LM | LV | LV | LM | LM | LM |
|  | A5 | LM | LM | LS | LS | LS | LM | LM | LS | LS | NT | LM | LM | LS | LS | NT | LM | LM | LS | LS | NT | LM | LM | LS | LS | NT |
|  | A6 | LV | LV | LM | LM | LM | LV | LM | LM | LM | LS | LV | LV | LM | LM | LM | LV | LM | LM | LS | LS | LV | LM | LM | LM | LS |
|  | A7 | LV | LM | LM | LS | LS | LM | LM | LS | LS | NT | LM | LM | LM | LS | LS | LM | LM | LM | LS | LS | LM | LM | LM | LS | LS |
|  | A8 | LM | LM | LM | LS | LS | LM | LM | LM | LS | LS | LM | LM | LM | LS | NT | LM | LM | LS | LS | NT | LM | LM | LS | LS | NT |
|  | A9 | LM | LM | LS | LS | NT | LM | LS | LS | LS | NT | LM | LS | LS | NT | NT | LM | LM | LS | LS | LS | LM | LM | LS | LS | LS |
|  | A10 | LV | LM | LM | LS | LS | LV | LM | LM | LS | LS | LM | LM | LS | LS | NT | LV | LM | LM | LS | LS | LV | LM | LM | LS | LS |
| P5 | A1 | LM | LS | LS | NT | NT | LM | LS | NT | NT | DS | LM | LM | LS | LS | NT | LM | LS | NT | NT | DS | LM | LS | LS | NT | NT |
|  | A2 | LM | LM | LS | LS | LS | LM | LM | LM | LS | LS | LM | LM | LM | LS | NT | LM | LM | LM | LS | LS | LM | LM | LS | LS | LS |
|  | A3 | LM | LM | LS | LS | NT | LM | LM | LM | LS | LS | LM | LM | LS | LS | NT | LM | LM | LS | LS | NT | LM | LM | LM | LS | LS |
|  | A4 | LV | LM | LM | LM | LM | LV | LV | LM | LM | LS | LV | LV | LV | LM | LM | LV | LM | LM | LM | LS | LV | LV | LM | LM | LM |
|  | A5 | LM | LM | LS | LS | LS | LM | LM | LM | LS | LS | LM | LM | LS | LS | NT | LM | LM | LS | LS | LS | LM | LM | LM | LS | LS |
|  | A6 | LV | LV | LM | LM | LM | LV | LV | LM | LM | LS | LV | LV | LM | LM | LS | LV | LV | LM | LM | LS | LV | LM | LM | LM | LM |
|  | A7 | LV | LM | LM | LS | LS | LV | LM | LS | LS | LS | LM | LS | LS | LS | NT | LM | LM | LM | LM | LS | LM | LM | LM | LS | LS |
|  | A8 | LM | LM | LS | LS | LS | LM | LM | LM | LS | LS | LM | LM | LM | LS | LS | LM | LM | LM | LS | LS | LM | LM | LS | LS | NT |
|  | A9 | LM | LM | LM | LM | LS | LM | LM | LS | LS | LS | LM | LS | LS | LS | LS | LV | LM | LM | LS | LS | LV | LM | LM | LS | LS |
|  | A10 | LM | LM | LS | LS | LS | LV | LM | LS | LS | NT | LM | LS | LS | LS | NT | LM | LM | LS | LS | LS | LM | LM | LS | LS | LS |
| P6 | A1 | LM | LS | LS | LS | NT | LM | LM | LS | LS | NT | LM | LM | LS | LS | NT | LM | LS | LS | NT | NT | LM | LS | LS | LS | NT |
|  | A2 | LM | LM | LS | LS | LS | LM | LM | LS | LS | LS | LM | LM | LM | LS | LS | LM | LM | LS | LS | NT | LM | LM | LS | LS | NT |
|  | A3 | LV | LM | LM | LS | LS | LM | LM | LS | LS | LS | LM | LS | LS | LS | NT | LM | LM | LM | LS | LS | LM | LM | LM | LS | LS |
|  | A4 | LV | LV | LM | LM | LM | LV | LV | LM | LM | LS | LV | LV | LM | LM | LS | LV | LM | LM | LM | LS | LV | LV | LM | LM | LM |
|  | A5 | LM | LM | LM | LS | LS | LM | LM | LM | LS | LS | LM | LM | LS | LS | LS | LM | LM | LM | LS | LS | LM | LM | LM | LM | LS |
|  | A6 | LV | LV | LM | LM | LS | LV | LM | LM | LM | LS | LV | LV | LM | LM | LS | LV | LV | LM | LM | LM | LV | LV | LM | LM | LM |
|  | A7 | LV | LM | LM | LM | LS | LM | LM | LS | LS | LS | LM | LM | LM | LS | LS | LM | LM | LS | LS | LS | LM | LM | LS | LS | LS |
|  | A8 | LM | LM | LM | LS | LS | LM | LS | LS | LS | LS | LV | LM | LM | LS | LS | LM | LM | LS | LS | NT | LM | LS | LS | LS | NT |
|  | A9 | LV | LM | LM | LM | LS | LM | LM | LM | LS | LS | LM | LM | LS | LS | NT | LM | LM | LS | LS | LS | LM | LM | LM | LS | LS |
|  | A10 | LM | LM | LS | LS | NT | LM | LM | LM | LS | LS | LM | LM | LS | LS | NT | LV | LM | LM | LS | LS | LV | LM | LM | LS | LS |
| P7 | A1 | LM | LS | LS | LS | NT | LM | LM | LS | LS | NT | LM | LM | LS | LS | NT | LM | LS | LS | LS | NT | LM | LS | LS | LS | LS |
|  | A2 | LM | LM | LS | LS | LS | LM | LM | LS | LS | LS | LM | LM | LS | LS | LS | LM | LM | LM | LS | LS | LM | LM | LM | LM | LS |
|  | A3 | LM | LM | LM | LS | LS | LM | LM | LM | LS | LS | LM | LM | LM | LM | LS | LV | LM | LM | LS | LS | LV | LV | LM | LM | LS |
|  | A4 | LV | LV | LM | LM | LS | LV | LM | LM | LM | LS | LV | LV | LM | LM | LS | LV | LV | LM | LM | LM | LV | LV | LV | LM | LM |
|  | A5 | LM | LM | LS | LS | LS | LM | LM | LS | LS | NT | LM | LM | LS | LS | NT | LM | LM | LS | LS | NT | LM | LM | LM | LS | NT |
|  | A6 | LV | LM | LM | LM | LS | LV | LV | LM | LM | LS | LM | LM | LM | LM | LS | LV | LM | LM | LM | LS | LV | LV | LM | LM | LM |
|  | A7 | LM | LM | LM | LS | LS | LM | LM | LS | LS | LS | LM | LM | LM | LS | LS | LM | LM | LM | LS | LS | LM | LM | LM | LS | LS |
|  | A8 | LM | LM | LM | LS | LS | LM | LM | LS | LS | NT | LM | LM | LS | LS | NT | LM | LM | LS | LS | NT | LM | LM | LM | LS | LS |
|  | A9 | LV | LM | LM | LS | LS | LV | LM | LM | LS | LS | LV | LV | LM | LM | LM | LV | LM | LS | LS | LS | LM | LM | LS | LS | LS |
|  | A10 | LV | LM | LM | LS | LS | LM | LM | LM | LS | LS | LM | LM | LM | LS | LS | LV | LM | LM | LM | LS | LV | LM | LM | LM | LS |

Legend: A1 – CS; A2 – MCC 0.5; A3 - MCC 1; A4 - MCC 1.5; A5 – MCP 0.5; A6 - MCP 1; A7 - MCP 1.5; A8 - MCCP 0.5 0.5; A9 - MCCP 1 1; A10 - MCCP 1.5 1.5

**Supplementary Table 3. Evolution of physicochemical parameters of melted cheese samples during the storage period**

| **Item** | **Samples** | **Storage period [days]** | | | | |
| --- | --- | --- | --- | --- | --- | --- |
|  |  | **1** | **15** | **30** | **45** | **60** |
| Acidity [°T] | CS | 57.97±0.02^a,A^ | 69.20±0.02^b,B^ | 92.12±0.02^c,C^ | 134.12±0.02^d,F^ | 157.18±0.02^e,F^ |
|  | MCC 0.5 | 60.96±0.01^a,B^ | 65.45±0.02^a,A^ | 76.47±0.02^b,A^ | 94.18±0.02^c,C^ | 111.13±0.02^d,D^ |
|  | MCC 1 | 64.97±0.02^a,B^ | 70.13±0.02^b,B^ | 80.14±0.02^c,B^ | 96.15±0.02^d,D^ | 109.18±0.01^e,C^ |
|  | MCC 1.5 | 67.97±0.03a,C | 71.86±0.01^b,B^ | 82.14±0.03^c,B^ | 97.05±0.02^d,D^ | 110.35±0.01^e,C^ |
|  | MCP 0.5 | 64.95±0.02^a,B^ | 68.22±0.01^a,AB^ | 75.37±0.02^b,A^ | 89.15±0.02^c,B^ | 106.14±0.02^d,B^ |
|  | MCP 1 | 65.96±0.02^a,BC^ | 68.96±0.01^a,AB^ | 76.13±0.02^b,A^ | 92.55±0.02^c,C^ | 107.39±0.01^d,B^ |
|  | MCP 1.5 | 66.99±0.01^a,BC^ | 70.02±0.02^b,B^ | 84.20±0.02^c,B^ | 97.76±0.02^d,D^ | 112.34±0.02^e,D^ |
|  | MCCP 0.5 0.5 | 60.02±0.02a,B | 66.12±0.02^a,A^ | 75.17±0.02^b,A^ | 86.25±0.02^c,A^ | 107.16±0.02^d,B^ |
|  | MCCP 1 1 | 76.98±0.01^a,D^ | 83.45±0.01^b,C^ | 91.14±0.03^c,C^ | 109.58±0.02^d,E^ | 123.12±0.02^e,E^ |
|  | MCCP 1.5 1.5 | 65.01±0.01^a,BC^ | 69.26±0.02^a,B^ | 78.35±0.03^b,AB^ | 88.24±0.03^c,B^ | 106.53±0.01^d,B^ |
|  | MCCP 1.5 1 | 65.99±0.02^a,BC^ | 69.45±0.02^a,B^ | 76.87±0.02^b,A^ | 84.01±0.02^c,A^ | 102.36±0.02^d,A^ |
| pH | CS | 6.15±0.001^c,C^ | 5.97±0.002^b,C^ | 5.12±0.002^b,A^ | 4.23±0.001^a,A^ | 4.01±0.001^a,A^ |
|  | MCC 0.5 | 6.12±0.002^c,C^ | 6.04±0.002^c,C^ | 5.79±0.002^bc,E^ | 5.09±0.002^ab,D^ | 4.76±0.002^a,D^ |
|  | MCC 1 | 6.06±0.002^c,B^ | 5.91±0.002^bc,B^ | 5.65±0.002^b,D^ | 5.00±0.002^a,C^ | 4.91±0.002^a,F^ |
|  | MCC 1.5 | 6.02±0.002^c,B^ | 5.89±0.002^bc,B^ | 5.57±0.002^b,D^ | 4.99±0.002^a,C^ | 4.82±0.001^a,E^ |
|  | MCP 0.5 | 6.06±0.002^d,B^ | 6.00±0.002^d,C^ | 5.82±0.002^c,F^ | 5.35±0.002^b,E^ | 5.00±0.001^a,G^ |
|  | MCP 1 | 6.04±0.001^c,B^ | 6.00±0.001^c,C^ | 5.80±0.002^b,F^ | 5.11±0.001^a,D^ | 4.99±0.001^a,G^ |
|  | MCP 1.5 | 6.04±0.002^d,B^ | 5.92±0.002^d,B^ | 5.48±0.001^c,C^ | 4.96±0.002^b,C^ | 4.54±0.002^a,C^ |
|  | MCCP 0.5 0.5 | 6.11±0.002^d,C^ | 6.01±0.002^d,C^ | 5.84±0.002^c,F^ | 5.51±0.002^b,F^ | 4.99±0.001^a,G^ |
|  | MCCP 1 1 | 5.77±0.001^d,A^ | 5.54±0.002^cd,A^ | 5.27±0.002^c,B^ | 4.88±0.002^b,B^ | 4.41±0.002^a,B^ |
|  | MCCP 1.5 1.5 | 6.07±0.002^d,B^ | 5.95±0.001^d,BC^ | 5.64±0.002^c,D^ | 5.38±0.002^b,E^ | 5.00±0.001^a,G^ |
|  | MCCP 1.5 1 | 6.04±0.001^d,B^ | 5.95±0.001^d,BC^ | 5.73±0.002^c,E^ | 5.47±0.002^b,F^ | 5.01±0.002^a,G^ |
| Water activity | CS | 0.935±0.001^a,F^ | 0.938±0.001^a,F^ | 0.943±0.001^b,F^ | 0.947±0.001^bc,E^ | 0.951±0.001^c,E^ |
|  | MCC 0.5 | 0.935±0.001^b,F^ | 0.934±0.001^b,E^ | 0.932±0.001^ab,D^ | 0.931±0.001^a,C^ | 0.929±0.001^a,C^ |
|  | MCC 1 | 0.933±0.001^c,D^ | 0.931±0.001^bc,B^ | 0.930±0.001^b,B^ | 0.929±0.001^b,A^ | 0.927±0.001^a,A^ |
|  | MCC 1.5 | 0.932±0.001^c,C^ | 0.931±0.001^bc,B^ | 0.929±0.001^b,A^ | 0.929±0.001^b,A^ | 0.927±0.001^a,A^ |
|  | MCP 0.5 | 0.935±0.001^c,F^ | 0.931±0.001^bc,B^ | 0.929±0.001^a,A^ | 0.929±0.001^a,A^ | 0.929±0.001^a,C^ |
|  | MCP 1 | 0.934±0.001^c,E^ | 0.931±0.001^bc,B^ | 0.930±0.001^b,B^ | 0.929±0.001^b,A^ | 0.928±0.001^a,B^ |
|  | MCP 1.5 | 0.933±0.001^c,D^ | 0.931±0.001^bc,B^ | 0.930±0.001^b,B^ | 0.929±0.001^b,A^ | 0.928±0.001^a,B^ |
|  | MCCP 0.5 0.5 | 0.933±0.001^d,D^ | 0.931±0.001^bc,B^ | 0.931±0.001^c,C^ | 0.930±0.001^b,B^ | 0.929±0.001^a,C^ |
|  | MCCP 1 1 | 0.942±0.001^c,G^ | 0.931±0.001^bc,B^ | 0.940±0.001^b,E^ | 0.939±0.001^b,D^ | 0.937±0.001^a,D^ |
|  | MCCP 1.5 1.5 | 0.931±0.001^c,B^ | 0.931±0.001^bc,B^ | 0.930±0.001^b,B^ | 0.929±0.001^b,A^ | 0.927±0.001^a,A^ |
|  | MCCP 1.5 1 | 0.930±0.001^c,A^ | 0.931±0.001^bc,B^ | 0.929±0.001^b,A^ | 0.929±0.001^b,A^ | 0.928±0.001^a,B^ |
| Dry matter content [%] | CS | 37.63±0.15^a,C^ | 40.93±0.15^b,E^ | 45.37±0.21^c,E^ | 52.67±0.15^d,F^ | 52.67±0.15^d,F^ |
|  | MCC 0.5 | 38.93±0.06^a,D^ | 39.27±0.15^b,D^ | 40.47±0.15^c,C^ | 42.33±0.15^d,C^ | 50.27±0.15^e,E^ |
|  | MCC 1 | 39.07±0.15^a,E^ | 39.63±0.12^a,D^ | 41.00±0.17^b,D^ | 42.87±0.06^c,C^ | 46.23±0.15^d,C^ |
|  | MCC 1.5 | 40.50±0.10^a,F^ | 40.93±0.06^a,E^ | 41.77±0.06^b,D^ | 44.13±0.06^c,E^ | 46.67±0.12^d,C^ |
|  | MCP 0.5 | 36.03±0.15^a,B^ | 37.87±0.15^b,B^ | 39.17±0.15^c,B^ | 41.43±0.15^d,B^ | 47.17±0.12^e,D^ |
|  | MCP 1 | 36.23±0.06^a,B^ | 38.63±0.12^b,C^ | 39.47±0.21^c,B^ | 41.77±0.12^d,B^ | 44.57±0.12^e,A^ |
|  | MCP 1.5 | 37.23±0.12^a,C^ | 39.10±0.10^b,D^ | 40.07±0.06^c,C^ | 42.63±0.12^d,C^ | 44.87±0.15^e,A^ |
|  | MCCP 0.5 0.5 | 37.33±0.15^a,C^ | 38.37±0.15^b,C^ | 39.73±0.15^c,B^ | 41.83±0.06^d,B^ | 46.43±0.06^e,C^ |
|  | MCCP 1 1 | 32.53±0.06^a,A^ | 32.93±0.06^a,A^ | 33.67±0.15^b,A^ | 36.07±0.06^c,A^ | 45.37±0.15^d,B^ |
|  | MCCP 1.5 1.5 | 39.33±0.015^a,E^ | 40.10±0.10^b,E^ | 40.80±0.10^b,C^ | 43.67±0.15^c,D^ | 44.93±0.15^b,A^ |
|  | MCCP 1.5 1 | 38.77±0.06^a,D^ | 39.57±0.15^b,D^ | 40.53±0.12^c,C^ | 43.23±0.15^d,D^ | 46.87±0.06^e,C^ |
| Salt content [g/100g product] | CS | 1.68±0.002^b,H^ | 1.68±0.001^b,G^ | 1.67±0.001^a,G^ | 1.67±0.001^a,H^ | 1.67±0.001^a,H^ |
|  | MCC 0.5 | 1.66±0.002^a,G^ | 1.66±0.001^a,F^ | 1.66±0.001^a,F^ | 1.66±0.001^a,G^ | 1.66±0.001^a,G^ |
|  | MCC 1 | 1.65±0.001^b,F^ | 1.64±0.001^a,E^ | 1.64±0.001^a,E^ | 1.64±0.001^a,E^ | 1.64±0.001^a,E^ |
|  | MCC 1.5 | 1.61±0.002^a,B^ | 1.61±0.001^a,B^ | 1.61±0.001^a,B^ | 1.61±0.001^a,B^ | 1.61±0.001^a,B^ |
|  | MCP 0.5 | 1.66±0.001^b,G^ | 1.66±0.001^b,F^ | 1.66±0.001^b,F^ | 1.65±0.001^a,F^ | 1.65±0.001^a,F^ |
|  | MCP 1 | 1.64±0.001^a,E^ | 1.64±0.001^a,E^ | 1.64±0.001^a,E^ | 1.64±0.001^a,E^ | 1.64±0.001^a,E^ |
|  | MCP 1.5 | 1.62±0.002^a,C^ | 1.62±0.001^a,C^ | 1.62±0.001^a,C^ | 1.62±0.001^a,C^ | 1.62±0.001^a,C^ |
|  | MCCP 0.5 0.5 | 1.66±0.001^a,G^ | 1.66±0.001^a,F^ | 1.66±0.001^a,F^ | 1.66±0.001^a,G^ | 1.66±0.001^a,G^ |
|  | MCCP 1 1 | 1.12±0.002^a,A^ | 1.12±0.001^a,A^ | 1.12±0.001^a,A^ | 1.12±0.001^a,A^ | 1.12±0.001^a,A^ |
|  | MCCP 1.5 1.5 | 1.62±0.001^a,C^ | 1.62±0.001^a,C^ | 1.62±0.001^a,C^ | 1.62±0.001^a,C^ | 1.62±0.001^a,C^ |
|  | MCCP 1.5 1 | 1.63±0.002^a,D^ | 1.63±0.001^a,D^ | 1.63±0.001^a,D^ | 1.63±0.001^a,D^ | 1.63±0.001^a,D^ |

Results are presented in form of mean ± standard deviation (n = 3). In each column and row, values with different lowercase and uppercase letters, respectively are significantly different (P<0.05)

CS: control sample; MCC 0.5: melted cheese with carrot peel (0.5 g : 100 g); MCC 1: melted cheese with carrot peel (1 g : 100 g); MCC 1.5: melted cheese with carrot peel (1.5 g : 100 g); MCP 0.5: melted cheese with potato peel (0.5 g : 100 g); MCP 1: melted cheese with potato peel (1 g : 100 g); MCP 1.5: melted cheese with potato peel (1.5 g : 100 g); MCCP 0.5 0.5: melted cheese with carrot peel (0.5 g : 100 g) and potato peel (0.5 g : 100 g); MCCP 1 1: melted cheese with carrot peel (1 g : 100 g) and potato peel (1 g : 100 g); MCCP 1.5 1.5: melted cheese with carrot peel (1.5 g : 100 g) and potato peel (1.5 g : 100 g); MCCP 1.5 1: melted cheese with carrot peel (1.5 g : 100 g) and potato peel (1 g : 100 g)

**Supplementary Table 4. Evolution of enzymatic parameters of melted cheese samples during the storage period**

| **Item** | **Samples** | **Storage period [days]** | | | | |
| --- | --- | --- | --- | --- | --- | --- |
|  |  | **1** | **15** | **30** | **45** | **60** |
| L-lactic acid content [g/100g product] | CS | 1.41±0.001^a,A^ | 1.43±0.001^b,B^ | 1.44±0.001^b,B^ | 1.46±0.001^c,D^ | 1.47±0.001^c,C^ |
|  | MCC 0.5 | 1.42±0.001^a,B^ | 1.42±0.001^a,A^ | 1.43±0.001^ab,A^ | 1.44±0.001^b,B^ | 1.45±0.001^b,B^ |
|  | MCC 1 | 1.42±0.001^a,B^ | 1.43±0.001^a,B^ | 1.43±0.001^a,A^ | 1.45±0.001^b,C^ | 1.45±0.001^b,B^ |
|  | MCC 1.5 | 1.42±0.001^a,B^ | 1.43±0.001^a,B^ | 1.43±0.001^a,A^ | 1.45±0.001^b,C^ | 1.45±0.001^b,B^ |
|  | MCP 0.5 | 1.42±0.001^a,B^ | 1.42±0.001^a,A^ | 1.43±0.001^ab,A^ | 1.44±0.001^b,B^ | 1.44±0.001^b,A^ |
|  | MCP 1 | 1.42±0.001^a,B^ | 1.43±0.001^ab,B^ | 1.43±0.001^ab,A^ | 1.44±0.001^b,B^ | 1.44±0.001^b,A^ |
|  | MCP 1.5 | 1.42±0.001^a,B^ | 1.43±0.001^ab,B^ | 1.44±0.001^b,B^ | 1.45±0.001^b,C^ | 1.45±0.001^b,B^ |
|  | MCCP 0.5 0.5 | 1.42±0.001^a,B^ | 1.42±0.001^a,A^ | 1.43±0.001^ab,A^ | 1.44±0.001^b,B^ | 1.44±0.001^b,A^ |
|  | MCCP 1 1 | 1.43±0.001^a,C^ | 1.44±0.001^ab,C^ | 1.44±0.001^ab,B^ | 1.45±0.001^b,C^ | 1.45±0.001^b,B^ |
|  | MCCP 1.5 1.5 | 1.42±0.001^a,B^ | 1.43±0.001^ab,B^ | 1.43±0.001^ab,A^ | 1.44±0.001^b,B^ | 1.44±0.001^b,A^ |
|  | MCCP 1.5 1 | 1.42±0.001^a,B^ | 1.43±0.001^ab,B^ | 1.43±0.001^ab,A^ | 1.43±0.001^ab,A^ | 1.44±0.001^b,A^ |
| Lactose content [g/100g product] | CS | 0.53±0.001^d,C^ | 0.52±0.001^c,B^ | 0.51±0.001^b,A^ | 0.49±0.001^a,A^ | 0.49±0.001^a,A^ |
|  | MCC 0.5 | 0.53±0.001^d,C^ | 0.52±0.001^c,B^ | 0.51±0.001^b,A^ | 0.51±0.001^b,C^ | 0.50±0.001^a,B^ |
|  | MCC 1 | 0.52±0.001^c,B^ | 0.52±0.001^c,B^ | 0.51±0.001^b,A^ | 0.50±0.001^a,B^ | 0.50±0.001^a,B^ |
|  | MCC 1.5 | 0.52±0.001^c,B^ | 0.52±0.001^c,B^ | 0.51±0.001^b,A^ | 0.50±0.001^a,B^ | 0.50±0.001^a,B^ |
|  | MCP 0.5 | 0.52±0.001^c,B^ | 0.52±0.001^c,B^ | 0.51±0.001^b,A^ | 0.51±0.001^b,C^ | 0.50±0.001^a,B^ |
|  | MCP 1 | 0.52±0.001^c,B^ | 0.52±0.001^c,B^ | 0.51±0.001^b,A^ | 0.51±0.001^b,C^ | 0.50±0.001^a,B^ |
|  | MCP 1.5 | 0.52±0.001^c,B^ | 0.52±0.001^c,B^ | 0.51±0.001^b,A^ | 0.50±0.001^a,B^ | 0.50±0.001^a,B^ |
|  | MCCP 0.5 0.5 | 0.52±0.001^c,B^ | 0.52±0.001^c,B^ | 0.51±0.001^b,A^ | 0.51±0.001^b,C^ | 0.50±0.001^a,B^ |
|  | MCCP 1 1 | 0.51±0.001^b,A^ | 0.51±0.001^b,A^ | 0.51±0.001^b,A^ | 0.50±0.001^a,B^ | 0.50±0.001^a,B^ |
|  | MCCP 1.5 1.5 | 0.52±0.001^c,B^ | 0.52±0.001^c,B^ | 0.51±0.001^b,A^ | 0.51±0.001^b,C^ | 0.50±0.001^a,B^ |
|  | MCCP 1.5 1 | 0.52±0.001^c,B^ | 0.52±0.001^c,B^ | 0.51±0.001^b,A^ | 0.51±0.001^b,C^ | 0.50±0.001^a,B^ |
| D-glucose content [g/100g product] | CS | 0.0023±0.00001^b,A^ | 0.0023±0.00001^b,A^ | 0.0023±0.00001^b,A^ | 0.0022±0.00001^a,A^ | 0.0022±0.00001^a,A^ |
|  | MCC 0.5 | 0.0023±0.00001^a,A^ | 0.0023±0.00001^a,A^ | 0.0023±0.00001^a,A^ | 0.0023±0.00001^a,B^ | 0.0023±0.00001^a,B^ |
|  | MCC 1 | 0.0023±0.00001^a,A^ | 0.0023±0.00001^a,A^ | 0.0023±0.00001^a,A^ | 0.0023±0.00001^a,B^ | 0.0023±0.00001^a,B^ |
|  | MCC 1.5 | 0.0024±0.00001^b,B^ | 0.0024±0.00001^b,B^ | 0.0023±0.00001^a,A^ | 0.0023±0.00001^a,B^ | 0.0023±0.00001^a,B^ |
|  | MCP 0.5 | 0.0023±0.00001^a,A^ | 0.0023±0.00001^a,A^ | 0.0023±0.00001^a,A^ | 0.0023±0.00001^a,B^ | 0.0023±0.00001^a,B^ |
|  | MCP 1 | 0.0024±0.00001^b,B^ | 0.0024±0.00001^b,B^ | 0.0023±0.00001^a,A^ | 0.0023±0.00001^a,B^ | 0.0023±0.00001^a,B^ |
|  | MCP 1.5 | 0.0024±0.00001^b,B^ | 0.0024±0.00001^b,B^ | 0.0024±0.00001^b,B^ | 0.0023±0.00001^a,B^ | 0.0023±0.00001^a,B^ |
|  | MCCP 0.5 0.5 | 0.0023±0.00001^a,A^ | 0.0023±0.00001^a,A^ | 0.0023±0.00001^a,A^ | 0.0023±0.00001^a,B^ | 0.0023±0.00001^a,B^ |
|  | MCCP 1 1 | 0.0024±0.00001^b,B^ | 0.0024±0.00001^b,B^ | 0.0023±0.00001^a,A^ | 0.0023±0.00001^a,B^ | 0.0023±0.00001^a,B^ |
|  | MCCP 1.5 1.5 | 0.0024±0.00001^b,B^ | 0.0023±0.00001^a,A^ | 0.0023±0.00001^a,A^ | 0.0023±0.00001^a,B^ | 0.0023±0.00001^a,B^ |
|  | MCCP 1.5 1 | 0.0024±0.00001^b,B^ | 0.0024±0.00001^b,B^ | 0.0023±0.00001^a,A^ | 0.0023±0.00001^a,B^ | 0.0023±0.00001^a,B^ |
| D-galactose content [g/100g product] | CS | 0.0423±0.00001^e,A^ | 0.0421±0.00001^d,A^ | 0.0420±0.00001^c,A^ | 0.0418±0.00001^b,A^ | 0.0414±0.00001^a,A^ |
|  | MCC 0.5 | 0.0425±0.00001^e,B^ | 0.0424±0.00001^d,B^ | 0.0423±0.00001^c,B^ | 0.0422±0.00001^b,B^ | 0.0421±0.00001^a,B^ |
|  | MCC 1 | 0.0426±0.00001^e,C^ | 0.0425±0.00001^d,C^ | 0.0424±0.00001^c,C^ | 0.0422±0.00001^b,B^ | 0.0421±0.00001^a,B^ |
|  | MCC 1.5 | 0.0428±0.00001^e,E^ | 0.0426±0.00001^d,D^ | 0.0424±0.00001^c,C^ | 0.0423±0.00001^b,C^ | 0.0422±0.00001^a,C^ |
|  | MCP 0.5 | 0.0426±0.00001^d,C^ | 0.0425±0.00001^c,C^ | 0.0423±0.00001^b,B^ | 0.0423±0.00001^b,C^ | 0.0421±0.00001^a,B^ |
|  | MCP 1 | 0.0428±0.00001^e,E^ | 0.0427±0.00001^d,E^ | 0.0425±0.00001^c,D^ | 0.0424±0.00001^b,D^ | 0.0423±0.00001^a,D^ |
|  | MCP 1.5 | 0.0428±0.00001^e,E^ | 0.0427±0.00001^d,E^ | 0.0426±0.00001^c,E^ | 0.0425±0.00001^b,E^ | 0.0424±0.00001^a,E^ |
|  | MCCP 0.5 0.5 | 0.0426±0.00001^e,C^ | 0.0425±0.00001^d,C^ | 0.0423±0.00001^c,B^ | 0.0422±0.00001^b,B^ | 0.0421±0.00001^a,B^ |
|  | MCCP 1 1 | 0.0427±0.00001^e,D^ | 0.0426±0.00001^d,D^ | 0.0425±0.00001^c,D^ | 0.0423±0.00001^b,C^ | 0.0422±0.00001^a,C^ |
|  | MCCP 1.5 1.5 | 0.0429±0.00001^e,F^ | 0.0427±0.00001^d,E^ | 0.0426±0.00001^c,E^ | 0.0425±0.00001^b,E^ | 0.0424±0.00001^a,E^ |
|  | MCCP 1.5 1 | 0.0428±0.00001^d,E^ | 0.0426±0.00001^c,D^ | 0.0425±0.00001^b,D^ | 0.0424±0.00001^a,D^ | 0.0424±0.00001^a,E^ |

Results are presented in form of mean ± standard deviation (n = 3). In each column and row, values with different lowercase and uppercase letters, respectively are significantly different (P<0.05)

CS: control sample; MCC 0.5: melted cheese with carrot peel (0.5 g : 100 g); MCC 1: melted cheese with carrot peel (1 g : 100 g); MCC 1.5: melted cheese with carrot peel (1.5 g : 100 g); MCP 0.5: melted cheese with potato peel (0.5 g : 100 g); MCP 1: melted cheese with potato peel (1 g : 100 g); MCP 1.5: melted cheese with potato peel (1.5 g : 100 g); MCCP 0.5 0.5: melted cheese with carrot peel (0.5 g : 100 g) and potato peel (0.5 g : 100 g); MCCP 1 1: melted cheese with carrot peel (1 g : 100 g) and potato peel (1 g : 100 g); MCCP 1.5 1.5: melted cheese with carrot peel (1.5 g : 100 g) and potato peel (1.5 g : 100 g); MCCP 1.5 1: melted cheese with carrot peel (1.5 g : 100 g) and potato peel (1 g : 100 g)

# Supplementary Figures


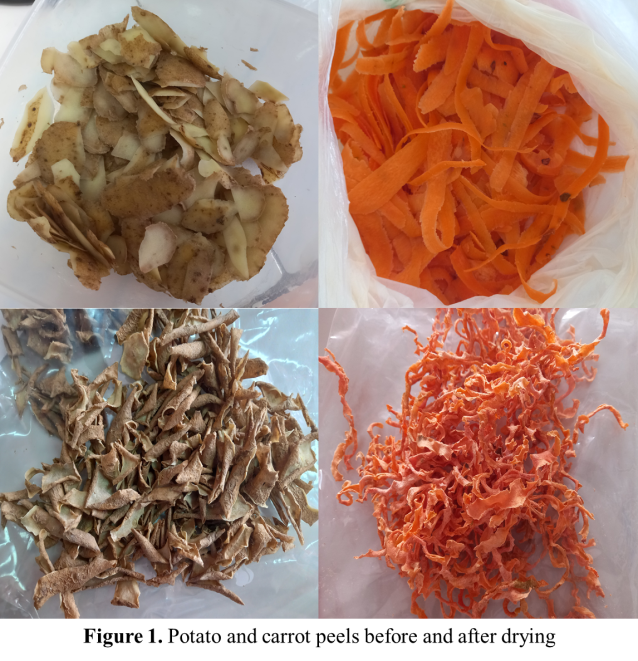


**Supplementary Figure 1.** Potato and carrot peels before and after drying
